# The Worldwide Spread of the Tiger Mosquito as Revealed by Mitogenome Haplogroup Diversity

**DOI:** 10.3389/fgene.2016.00208

**Published:** 2016-11-23

**Authors:** Vincenza Battaglia, Paolo Gabrieli, Stefania Brandini, Marco R. Capodiferro, Pio A. Javier, Xiao-Guang Chen, Alessandro Achilli, Ornella Semino, Ludvik M. Gomulski, Anna R. Malacrida, Giuliano Gasperi, Antonio Torroni, Anna Olivieri

**Affiliations:** ^1^Dipartimento di Biologia e Biotecnologie “L. Spallanzani”, Università di PaviaPavia, Italy; ^2^Crop Protection Cluster, College of Agriculture, University of the Philippines Los BañosLos Baños, Philippines; ^3^Department of Pathogen Biology, School of Public Health and Tropical Medicine, Southern Medical UniversityGuangzhou, China

**Keywords:** *Aedes albopictus*, tiger mosquito, mitochondrial DNA, mitogenomes, haplogroups

## Abstract

In the last 40 years, the Asian tiger mosquito *Aedes albopictus*, indigenous to East Asia, has colonized every continent except Antarctica. Its spread is a major public health concern, given that this species is a competent vector for numerous arboviruses, including those causing dengue, chikungunya, West Nile, and the recently emerged Zika fever. To acquire more information on the ancestral source(s) of adventive populations and the overall diffusion process from its native range, we analyzed the mitogenome variation of 27 individuals from representative populations of Asia, the Americas, and Europe. Phylogenetic analyses revealed five haplogroups in Asia, but population surveys appear to indicate that only three of these (A1a1, A1a2, and A1b) were involved in the recent worldwide spread. We also found out that a derived lineage (A1a1a1) within A1a1, which is now common in Italy, most likely arose in North America from an ancestral Japanese source. These different genetic sources now coexist in many of the recently colonized areas, thus probably creating novel genomic combinations which might be one of the causes of the apparently growing ability of *A. albopictus* to expand its geographical range.

## Introduction

The genus *Aedes* includes five highly invasive species, *A. albopictus, A. aegypti, A. j. japonicus, A. koreicus*, and *A. atropalpus*. Of these, *A. albopictus* and *A. j. japonicus* are the most widespread across the globe, and *A. aegypti* and *A. albopictus*, being competent vectors for several human tropical diseases, have a major impact on human health. The North American species, *A. atropalpus*, arrived in Europe (Italy, France, and the Netherlands) through international trade (Romi et al., 1997), but it was subsequently exterminated in Italy and France and is unlikely to have established in the Netherlands due to climatic conditions (Scholte et al., 2009). *Aedes j. japonicus* and *A. koreicus*, native to East Asia (Japan, Korea, China, Russia), have both colonized central Europe and *A. j. japonicus* is also widely distributed in the US (Medlock et al., 2015). *Aedes aegypti* originated in sub-Saharan Africa and is now considered the main vector of dengue. Climate appears to be the decisive factor limiting the distribution of *A. aegypti* to tropical and sub-tropical regions, with few incursions into Europe and North America (Powell and Tabachnick, 2013; Khormi and Kumar, 2014).

In contrast, *A. albopictus*, indigenous to East Asia, is not so restrained by climatic factors, and in the last 40 years has successfully colonized the tropical and temperate regions of all continents (Benedict et al., 2007; Paupy et al., 2009; Bonizzoni et al., 2013; Kraemer et al., 2015). This mosquito has become a growing public health concern, being a competent vector for many arboviruses which cause lethal or debilitating human diseases, including the dengue (DEN), chikungunya (CHIK), West Nile (WN) viruses (Gasperi et al., 2012; Bonizzoni et al., 2013), and the recently emerged ZIKA virus (Wong et al., 2013; Chouin-Carneiro et al., 2016). Although it has been considered a less efficient vector than *A. aegypti*, this species is the sole vector of recent DENV outbreaks in Southern China, Hawaii, the Indian Ocean and Gabon and the first autochthonous DENV transmission in France and Croatia (Paupy et al., 2009; Wu et al., 2010; Peng et al., 2012; Rezza, 2012, 2014; Schaffner et al., 2013). *A. albopictus* was most likely the main DENV vector in Asia prior to the introduction of *A. aegypti* in the mid nineteenth century (Gubler, 2006).

Introduced into Europe (Albania) in 1979, *A. albopictus* has now colonized all Mediterranean countries from Spain to Syria and has been reported in Central Europe (Medlock et al., 2012). It was introduced into Hawaii at the end of the 19th century (Rai, 1991) and into continental USA (Texas) in 1985, and is now well established in 32 states. Its presence has been reported in Mexico (first recorded in 1988), Central and South America (first recorded in Brazil, 1986), and Africa (first recorded in South Africa, 1989; Bonizzoni et al., 2013). Its ability to spread from the native range and adapt to local environments is probably due to its ecological characteristics, drought-resistant eggs with the ability to diapause, daylight biting habit, aggressive and opportunistic feeding behavior, and capacity to achieve high population densities (Paupy et al., 2009).

The nuclear genome of the tiger mosquito from two laboratory strains, the Italian Fellini (an isofemale line derived from the Rimini strain; Bellini et al., 2007; Dritsou et al., 2015) and the Chinese Foshan strain (Chen et al., 2015), was recently published, but only two complete mitogenomes (∼16.7 kb) are available in GenBank, from Taiwan (Asian tiger mosquito Reference Sequence, NC006817) and from Nanjing, Jiangsu Province, China (KR068634; Zhang et al., 2015). Despite the availability of mitogenomes, virtually all *A. albopictus* mitochondrial DNA (mtDNA) surveys were restricted to short segments of the cytochrome c oxidase subunit 1 (COI) and/or NADH dehydrogenase subunit 5 (ND5) genes, suggesting a limited phylogeographic differentiation among populations, possibly also caused by the inclusion in these studies of laboratory stocks or sibling eggs (Delatte et al., 2011; Kamgang et al., 2011, 2013; Porretta et al., 2012; Zhong et al., 2013; Zawani et al., 2014; Futami et al., 2015) and the postulated cytoplasmatic sweep caused by *Wolbachia* infection (Armbruster et al., 2003). However, more extensive sequencing of the COI gene has revealed more variation than previously thought (Goubert et al., 2016), a scenario also partially supported by microsatellite studies that highlighted slight genetic diversity between native and adventive populations with high variability within populations (Manni et al., 2015).

Previous studies have shown that the variation seen in short mtDNA segments may be inadequate to both identify and phylogenetically link haplogroups (Torroni et al., 2006; Achilli et al., 2008, 2012). This is especially true for the insect mtDNA control region due to its peculiar features: high A+T content and reduced substitution rate, variable size and high length mutation rate, concerted evolution of tandem repeats and directional mutation pressure (Zhang and Hewitt, 1997).

To identify the ancestral source(s) of *A. albopictus* adventive populations, overcoming previous limitations, we here determined and analyzed the sequence variation at the level of entire coding regions of 27 mitogenomes (25 novel and two previously published) from Eastern and Southeastern Asian, American, and European populations. Our analyses reveal that only three of the five identified Asian haplogroups, which are differentially distributed in Asian populations living in temperate and tropical regions, were involved in the recent worldwide spread. These different ancestral sources from Asia now coexist in many adventive populations with possible implications for the adaptive capability of the species.

## Materials and Methods

### Sample Collection and DNA Extraction

**FIGURE 1:**
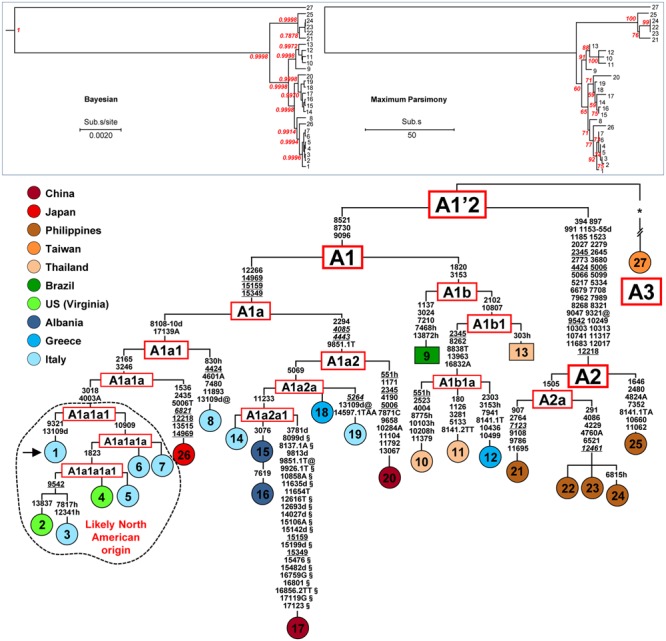
**Phylogeny of *A. albopictus* mitogenomes.** The Bayesian (left) and MP (right) trees are shown in the top inset. The posterior probability for the major nodes in the Bayesian tree is reported in red, whereas bootstrap values (1000 replications) are reported in red in the MP tree. These trees encompass 25 novel and two previously published sequences (**Table 1**). A magnified MP tree is also shown reporting all mutations that characterize the 27 mitogenomes except those linking mitogenome #27 to the A1′2 node (see below). The asterisk (^∗^) indicates the location of these mutations (347 in the coding region and 16 in control region), which are listed in Supplementary information for Figure 1. For the phylogeny construction, the entire coding region variation of all mitogenomes was included as well as some control region mutations (see “Material and Methods”). The published sequences (#17 and #27, **Table 1**) are from the Jiangsu Province, China (Zhang et al., 2015) and from Taipei, Taiwan, respectively. The mitogenome from a mosquito of the Italian Rimini strain (#1, marked by the arrow) was employed to number the mutations shown on the branches. Mutations are transitions unless a base is explicitly indicated for transversions (to A, G, C, or T) or a suffix for indels (0.1, d). Heteroplasmic positions are marked by an “h.” Recurrent mutations within the phylogeny are underlined (and in italics if present in mitogenome #27) and back mutations are marked with the suffix @. The numerous mutations shared only by the published mitogenomes #17 and #27 are marked with the suffix §. Taking also into account that, despite their extensive coding region differentiation, mitogenomes #17 and #27 intriguingly harbor virtually identical control region sequences, it is likely that at least some of the mutations marked with the suffix § are mistakes. Colors illustrate geographic origins. **Table 1** provides additional information concerning the geographic origin and haplogroup affiliation of each sample. Length variation (insertions/deletions) in a poly-A stretch beginning at np 3808 was not considered. Note that for mitogenomes #9 and #13 (in squared boxes) the sequence variation was assessed only partially for the coding region and not at all for the control region (Supplementary information for Figure 1). The sub-haplogroup A1a1a1 and its derivatives of probable North American origin are encircled.

A total of 25 novel mitogenomes were included in this study. Twenty-two were from wild populations collected in Europe, Asia, and the Americas (**Table 1**; **Figure 1**). Three were from the Americas (two from Virginia and one from Brazil). Nine were from Asia: three from Thailand (one from Hang Chat district, Lampang province in the North; one from Ban Rai district, Uthai Thani province in the West; one from Phato district, Chumphon province in the South), five from Los Baños, Laguna, Philippines and one from Wakayama prefecture, Japan. Ten were from Europe: two from Tirana (Albania), two from Athens (Greece), two from Cesena and two from Pavia (Northern Italy), one from Cassino (Central Italy), and one from Reggio Calabria (Southern Italy). This study also included three adult laboratory-maintained strain mosquitoes: two from the Italian Rimini strain (Bellini et al., 2007; Manni et al., 2015), established at CAA (Centro Agricoltura Ambiente “G. Nicoli,” Crevalcore, Italy) from mosquitoes collected in Rimini, Italy, and one from the Chinese Foshan strain (Center for Disease Control and Prevention of Guangdong Province; **Table 1**). The study did not involve protected species and specimens were not collected at sites protected by law.

**Table 1:** Origin and haplogroup affiliation of *A. albopictus* mitogenomes considered in this study.

Sequence ID#^a^	Original name	Continent	Country (place of collection)	Haplogroup	GenBank ID	Number of type I repeats^b^	Number of type II repeats^b^	Reference
1	Rim1^c^	Europe	Italy, Rimini	A1a1a1	KX383916	7	5	this study
2	Vir1	America	US, Virginia	A1a1a1a1	KX383917	N.D.	6	this study
3	Rc1	Europe	Italy, Reggio Calabria	A1a1a1a1	KX383918	N.D.	6	this study
4	Vir2	America	US, Virginia	A1a1a1a1	KX383919	N.D.	6	this study
5	Ces1	Europe	Italy, Cesena	A1a1a1a1	KX383920	N.D.	6	this study
6	Cas1	Europe	Italy, Cassino	A1a1a1a	KX383921	N.D.	6	this study
7	Pav3	Europe	Italy, Pavia	A1a1a1a	KX383922	N.D.	6	this study
8	Ces2	Europe	Italy, Cesena	A1a1	KX383923	N.D.	4	this study
9	Bra	America	Brazil	A1b	KX383924	N.D.	N.D.	this study
10	Lam2	Asia	Thailand, Lampang, Hang Chat	A1b1a	KX383925	N.D.	3	this study
11	Ban7	Asia	Thailand, Uthai Thani, Ban Rai	A1b1a	KX383926	N.D.	3	this study
12	Ath1	Europe	Greece, Athens	A1b1a	KX383927	N.D.	3	this study
13	Chu3	Asia	Thailand, Chumphon, Phato	A1b1	KX383928	N.D.	3	this study
14	Rim4^c^	Europe	Italy, Rimini	A1a2a1	KX383929	N.D.	4	this study
15	Tir1	Europe	Albania, Tirana	A1a2a1	KX383930	N.D.	4	this study
16	Tir2	Europe	Albania, Tirana	A1a2a1	KX383931	N.D.	4	this study
17	–	Asia	China, Jiangsu, Nanjing	A1a2a1	KR068634	5	4	Zhang et al., 2015
18	Ath2	Europe	Greece, Athens	A1a2a	KX383932	N.D.	4	this study
19	Pav4	Europe	Italy, Pavia	A1a2a	KX383933	N.D.	4	this study
20	Fo2^c^	Asia	China, Foshan	A1a2	KX383934	N.D.	4	this study
21	Los1	Asia	Philippines, Laguna, Los Baños	A2a	KX383935	N.D.	3	this study
22	Los2	Asia	Philippines, Laguna, Los Baños	A2a	KX809761	N.D.	3	this study
23	Los3	Asia	Philippines, Laguna, Los Baños	A2a	KX809762	N.D.	3	this study
24	Los5	Asia	Philippines, Laguna, Los Baños	A2a	KX809764	N.D.	3	this study
25	Los4	Asia	Philippines, Laguna, Los Baños	A2	KX809763	N.D.	3	this study
26	J-Wa1	Asia	Japan, Wakayama	A1a1a	KX809765	N.D.	4	this study
27	–	Asia	Taiwan, Taipei	A3	NC006817	5	4	–

*^a^ID numbers correspond to those in **Figure 1**.*

*^b^N.D. = not determined.*

*^c^Laboratory-maintained strain.*

Morphological keys (Rueda, 2004) and/or PCRs with species-specific primers for internal transcribed spacer regions (ITS1 and ITS2) of ribosomal DNA (rRNA; Higa et al., 2010) were used to identify the specimens. For the Philippine samples, eggs were collected using ovitraps, and the emerging adults were reared in an insectary under standard conditions of temperature (27°C), humidity (60–80%), and photoperiod (12:12 h). Samples were preserved in 80% ethanol and stored at -20°C until DNA extraction. DNA extraction was carried out using the Wizard Genomic DNA Purification Kit (Promega) following the manufacturer’s protocol.

### Sequencing of the *A. albopictus* Mitochondrial Coding and Control Regions

A primer set was designed to amplify the entire *A. albopictus* mitogenome in four overlapping PCR fragments (protocol I). The *A. albopictus* Reference Sequence (NC006817) was used to design primer sets. The coding region (nps 1-14893) was amplified by two long PCR fragments whereas the control region (nps 14894-16665) was amplified by two rather short PCR fragments (Supplementary Table 1) because of its high A+T content and the presence of repeated elements requiring distinctive PCR procedures.

Coding region long PCRs (Supplementary Table 1) were carried out in 50 μl reaction mixture containing 1X GoTaq Long PCR Master Mix (Promega), 0.2 μM of each primer and 10–20 ng of DNA template using the following PCR conditions: 94°C (2 min); 30 cycles of 94°C (30 s), 59°C (30 s), 65°C (9 min); and a final extension of 72°C (10 min). Alternatively, a set of nine overlapping PCR fragments (Supplementary Table 2) covering the *A. albopictus* coding region was also designed (protocol II). PCRs were carried out in 25 μl reaction with a standard reaction mix containing 1X White Buffer (1.5 mM MgCl_2_), 0.2 mM of each dNTP mix, 0.6 U of GoTaq G2 Polymerase (Promega), 0.2 μM of each primer and 20–30 ng of DNA template, using the following PCR conditions: 94°C (2 min); 35 cycles of 94°C (30 s), 55°C (30 s), 72°C (2 min); and a final extension of 72°C (5 min).

PCR primers used to amplify the control region in two overlapping fragments (Supplementary Table 1) were the same in both protocols. The control region PCRs were carried out using the following PCR conditions: 94°C (2 min); 35 cycles of 94°C (30 s), 54°C (30 s), 60°C (2 min); and a final extension of 60°C (5 min) for PCR #3, and 94°C (2 min); 40 cycles of 94°C (30 s), 55°C (30 s), 60°C (1 min); and a final extension of 60°C (10 min) for PCR #4. PCR products were visualized on a 1–2% agarose gel and successful amplicons were sequenced with standard dideoxy sequencing using Big Dye v3.1 Chemistry (Applied Biosystems) on 3730xl and 3130xl Genetic Analyzer (Applied Biosystems) following the manufacturer’s protocol. Sets of 28 and 29 oligonucleotides were designed to sequence the *A. albopictus* mtDNA coding region starting from protocol I (Supplementary Table 3) or protocol II (Supplementary Table 4), respectively. Sequences were assembled and aligned using Sequencher 5.0 (Gene Codes) comparing them with the Reference Sequence (NC006817) from a Taipei sample, Taiwan.

### Cloning and Sequencing of Mitogenome #1 (Rimini Strain) Control Region

**FIGURE 2:**
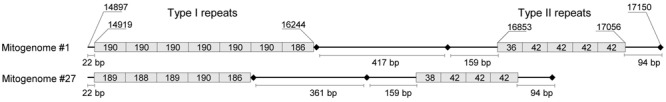
**Organization of tandem repeats in the control region of *A. albopictus* mitogenomes.** This schematic representation is based on the complete sequence information of mitogenomes #1 (Rimini strain) and #27 (**Figure 1**; **Table 1**). Two types of repeats (I and II) were observed with copy number differing for both repeats in the two mitogenomes. Type I consists of a ∼190 bp repeat unit whereas type II is made up of a shorter unit of ∼42 bp. The exact length (in bp) of each repeat is reported inside the box. The size of boxes representing repeats is not proportional to repeat lengths. Shown nucleotide positions are relative to sequence #1. Variation in size due to variable number of tandem repeats in the two regions, when available, is provided in **Table 1**. Black diamonds represent in the order from left to right: a poly-T stretch motif, a poly-T stretch motif followed by a GC-rich block, and a short poly-A stretch motif. All of these are conserved in different mosquito species (Dueñas et al., 2006).

Given the different copy numbers of repeated elements contained in the *A. albopictus* control region, PCR fragments #3 and #4 (Supplementary Table 1) yielded products of different length in different mosquitoes. The two types of tandem repeats, I and II (**Figure 2**), were amplified with PCR #3 and PCR #4, respectively. Overall, the size of the amplified fragments ranged from ∼1,800 to ∼2,500 bp for PCR #3 and from ∼800 to ∼900 bp for PCR #4. As for one of the two mitogenomes from the Rimini strain (mitogenome #1, **Figure 1**; **Table 1**), PCR #3 and #4 yielded products of ∼2,000 bp and ∼900 bp, respectively. The product of PCR #4 was directly cloned in the pCR2.1 TOPO vector (Invitrogen) following the manufacturer’s protocol. White colonies were PCR-screened for the insert length and desired clones were sequenced bi-directionally using the M13 universal primers. The procedure described above is inefficient for longer fragments such as that of ∼2,000 bp, therefore a different strategy was developed to sequence the region amplified by PCR #3. It was re-amplified in two overlapping PCR fragments (PCRs I and II), each with one primer within the tandemly repeated elements of type I (Supplementary Table 5). Multiple amplicons were obtained for each PCR and were cloned as described above. Only the two clones containing the longest fragments deriving from each PCR were sequenced bi-directionally. Sequences for each clone were assembled and aligned using Sequencher 5.0 (Gene Codes). Two additional internal primers were then newly designed (data not shown) to confirm the sequence obtained by cloning.

### Phylogeny Construction

The obtained complete sequence of mitogenome #1 (accession number KX383916) was used to assemble and number, with Sequencher 5.0 (Gene Codes), the other 24 novel mitogenomes (accession numbers KX383917-35, KX809761-65). We aligned the novel sequences and the two previously published mitogenomes – #17 from China (Zhang et al., 2015) and #27 from Taiwan (NC006817) – by performing a Multiple-Sequence Alignment with the Clustal algorithm (Chenna et al., 2003) implemented by Sequencher 5.0.

Most Parsimonious (MP) trees (1000 bootstrap replications) encompassing the 27 mitogenomes were built by using MEGA7 (Kumar et al., 2016), employing the Tree-Bisection-Regrafting (TBR) algorithm (Nei and Kumar, 2000). Modelgenerator v.85 indicated for our dataset HKY+I as the best-supported model according to the AIC1, AIC2, and BIC criteria. The obtained settings were selected to infer maximum likelihood (ML) and Bayesian trees for the *A. albopictus* dataset. The ML tree was built using PAMLX (Yang, 2007) and assuming the HKY85 mutation model (two parameters in the model of DNA evolution) with gamma-distributed rates (approximated by a discrete distribution with eight categories). The Bayesian tree was obtained using BEAST 1.8.3 (Drummond and Rambaut, 2007) and running 50,000,000 iterations, with samples drawn every 10,000 Markov chain Monte Carlo (MCMC) steps. It was visualized using FigTree v.1.4.2. Phylogeny reconstruction was performed considering all the nucleotide substitutions (excluding indels and heteroplasmies) in the coding region (from np 1 to np 14896, relative to mitogenome #1) and the five informative control region mutations 14969, 15159, 15349, 16832A, and 17139A. Thirteen additional control region mutations (see Supplementary information for Figure 1), ten shared exclusively by the previously published mitogenomes #17 and #27 and whose reliability is doubtful, and three (private mutations) seen only in mitogenome #27, were simply superimposed on the magnified MP tree (**Figure 1**).

### Survey of Published Partial COI and ND5 mtDNA Sequences

The identification of haplogroup diagnostic mutations allowed us to survey 1170 *A. albopictus* partial COI and ND5 available in the literature for the presence/absence of these mutations. Of these, 284 mtDNAs are from Asia, 349 from the Americas, 32 from Europe, 153 from Africa, and 352 from Oceania (**Table 2**), thus encompassing populations from both native and non-native areas, and living at different climatic conditions.

**Table 2:** Frequencies of *A. albopictus* mtDNA haplogroups in worldwide populations.

*Geographic origin*	*N*	Haplogroup frequencies^a^						Reference
		A1a1a1	A1a2	A1b	A2	A3	Others^b^	
***America***	349	94 (*0.27*)	59 (*0.17*)	141 (*0.40*)	0	0	55 (*0.16*)	
USA (New Jersey)	30	28 *(0.93*)	0	0	0	0	2 (*0.07*)	Zhong et al., 2013
USA (California)	49	0	29 (*0.59*)	5 (*0.10*)	0	0	15 (*0.31*)	Zhong et al., 2013
USA (Texas)	31	4 (*0.77*)	0	2 (*0.07*)	0	0	5 (*0.16*)	Zhong et al., 2013
USA (Hawaii)	32	0	27 (*0.84*)	0	0	0	5 (*0.16*)	Zhong et al., 2013
Costa Rica	57	29 (*0.51*)	0	0	0	0	28 (*0.49*)	Futami et al., 2015
Panama	16	13 (*0.81*)	*3 (0.19)*	0	0	0	0	Futami et al., 2015
Brazil^c^	134	0	0	134 (*1.0*)	0	0	0	Birungi and Munstermann, 2002
***Europe***	32	10 (*0.31*)	20 (*0.63*)	0	0	0	2 (*0.06*)	
Italy (Trento)	32	10 (*0.31*)	20 (*0.63*)	0	0	0	2 (*0.06*)	Zhong et al., 2013
***Africa***	153	0	0	153 (*1.0*)	0	0	0	
Cameroon	153	0	0	153 (*1.0*)	0	0	0	Kamgang et al., 2011
***Asia***	284	0	100 (*0.35*)	102 (*0.36*)	25 (*0.09*)	0	57 (*0.20*)	
China^d^	61	0	39 (*0.64*)	0	0	0	22 (*0.36*)	Zhong et al., 2013
China^e^ (strain)	30	0	*23 (0.77*)	0	0	0	7 (*0.23*)	Zhong et al., 2013
Japan	15	0	15 (*1.00*)	0	0	0	0	Zhong et al., 2013
Taiwan	30	0	4 (*0.13*)	0	0	0	26 (*0.87*)	Zhong et al., 2013
Thailand	10	0	0	10 (*1.00*)	0	0	0	Sumruayphol et al., 2016
Malaysia	77	0	0	77 (*1.00*)	0	0	0	Zawani et al., 2014
Singapore	36	0	19 (*0.53*)	15 (*0.42*)	0	0	2 (*0.05*)	Zhong et al., 2013
Indonesia (Java)	8	0	0	0	8 (*1.00*)	0	0	Beebe et al., 2013
Indonesia (Timor-Leste)	17	0	0	0	17 (*1.00*)	0	0	Beebe et al., 2013
***Oceania***	352	0	0	236 (*0.67*)	115 (*0.33*)	0	1 *(<0.01*)	
Australia (Torres Strait)	115	0	0	42 (*0.36*)	72 (*0.63*)	0	1 (*0.01*)	Beebe et al., 2013
Papua New Guinea	170	0	0	*162 (0.95*)	8 (*0.05*)	0	0	Beebe et al., 2013
Papua New Guinea (Southern Fly)	67	0	0	32 (*0.48*)	35 (*0.52*)	0	0	Beebe et al., 2013

*^a^Haplogroup affiliation is based on the sequence variation of the cytochrome c oxidase subunit I (COI) gene, except for the Brazilian samples (see footnote c). The diagnostic mutations are the lack of the transitions at nps 2165 and 1536 for haplogroup A1a1a1, the presence of the transition at np 2294 for haplogroup A1a2, the presence of the transition at np 1820 for haplogroup A1b, and the presence of the transition at np 2027 for haplogroup A2.*

*^b^These samples were classified as “others” because they harbor the transition at np 2165 (except those from Australia, in which that nucleotide position was not sequenced), thus they do not belong to haplogroup A1a1a. Moreover, they lack the transitions at nps 2294, 1820, and 2027, thus they are not members of haplogroups A1a2, A1b, nor A2. Some additional mutations found in these samples are shared between specimens from the same or different geographic areas. In most cases they are likely due to multiple mutational events (recurrent mutations), but some might instead mark additional and not yet identified haplogroups.*

*^c^Samples from Brazil were classified on the basis of their NADH dehydrogenase subunit 5 (ND5) gene sequences. They were considered members of haplogroup A1b because they harbored the transition at np 7210.*

*^d^Samples are from Guangdong and Fujian provinces.*

*^e^Laboratory-maintained strain, Jiangsu province.*

## Results

### The Variation of *A. albopictus* mtDNA Control Region

The first step in our study was to sequence the entire mtDNA (mitogenome #1) from one mosquito of the Italian (Rimini) laboratory-maintained strain (**Table 1**; **Figure 1**), whose nuclear genome was recently sequenced (Dritsou et al., 2015). This mitogenome sequence confirmed that the *A. albopictus* control region belongs to group 2 of insect control regions (Zhang and Hewitt, 1997), like that of *A. aegypti* (Dueñas et al., 2006). Three conserved blocks are positioned along the region, which contains two different types (I and II) of tandem repeats (**Figure 2**). Type I consists of a ∼190 bp repeat unit, whereas type II is made up of a short unit of ∼42 bp. The number of type I and type II tandem repeats that we observed in mitogenome #1 was different from those in sequence NC006817 from Taiwan (mitogenome #27 in **Figures 1** and **2**) and variable among mtDNAs (**Table 1** and data not shown). Moreover, between these two types of repeats, delimited by two conserved blocks, lies an A+T rich region of variable length. The overall length and tandem repeat composition make the PCR amplification and sequencing of the entire *A. albopictus* control region extremely difficult (see “Material and Methods”). For the reasons outlined above, and difficulties originating from the impossibility of distinguishing heteroplasmy from PCR artifacts (due to replication slippage), we restricted our sequencing of *A. albopictus* mitogenomes to the coding region (from np 1 to np 14893, NC006817) and nearby control region segments. This approach was employed to obtain the coding region sequences of the additional 24 mitogenomes.

### MtDNA Haplogroups in *A. albopictus*

**Figure 1** illustrates the Bayesian and MP trees derived from the coding regions of the 27 *A. albopictus* mitogenomes (25 novel and two previously published). The overall tree structure is virtually identical with the two approaches and is supported by the ML tree (Supplementary Figure 1), indicating a high degree of internal consistency for all major branches. A magnified MP tree is shown in the lower part of **Figure 1** in order to illustrate the branch location of the identified mutations.

The mitogenomes (24 distinct haplotypes) cluster into three major branches that we named haplogroups A1, A2, and A3. Haplogroup A1 includes 21 mitogenomes, A2 consists of the five Philippine samples, while A3 encompasses only one mitogenome (#27) from Taiwan. Haplogroups A1 and A2 are rather close to each other and to the A1′2 node. In contrast, the A3 mitogenome differs by 363 mutations from the same node.

Overall, this phylogeny reveals an extensive and previously unreported mitogenome differentiation within *A. albopictus*. Indeed, when calculated on the standard COI sequence (Hebert et al., 2003) employed for DNA barcoding, the maximum intraspecific divergence was 0.012 (eight mutations in 658 bp), a value in line with those recently reported for *A. scutellaris* (0.008) and *A. aegypti* (0.022), but much greater than the value previously reported for *A. albopictus* (0.002; Sumruayphol et al., 2016).

Haplogroup A1, which encompasses most of the mitogenomes in the phylogeny, is subdivided into two branches that we termed A1a and A1b, with the former further split into A1a1 and A1a2. In our phylogeny these clades and subclades appear to be correlated with different geographic distributions. The branch A1a1 includes the single mitogenome from Japan, the two mitogenomes from the US (Virginia) and many of the mitogenomes from Italy, including one (#1) of the two detected in the Rimini laboratory strain. The mitogenome from Japan (#26) departs from the node A1a1a and its sister clade A1a1a1 contains a sub-branch, A1a1a1a1, of particular interest. It consists of four mitogenomes, two from Italy and the two from the US mentioned above. One of the US mitogenomes (#4) is identical to one from Northern Italy (#5) while the second (#2) is closely related to the mitogenome #3 from Southern Italy.

The sister branch A1a2 is formed by mitogenomes from different regions of Southern Europe (Italy, Albania, and Greece), including the second mitogenome (#14) from the Rimini strain, the previously published Chinese sequence [#17, KR068634 (Zhang et al., 2015)] and the mitogenome (#20) from the Chinese Foshan strain, a laboratory-maintained colony founded in 1981 from mosquitoes from Southeast China. It is worth mentioning that the presence of two distinct haplotypes in different subjects of the Rimini laboratory-maintained strain, one belonging to A1a1a1 and the second to A1a2a1, reveals that at least two females contributed to the genetic formation of the strain. Haplogroup A1b contains all the mitogenomes from Thailand, which cluster in its A1b1 sub-branch, as well as one from Greece and one from Brazil. Finally, haplogroup A2 consists of multiple haplotypes, all from the Philippines.

### Haplogroup Affiliation of Worldwide mtDNA Sequences from *A. albopictus*

The phylogenetic analysis not only allowed the identification of *A. albopictus* haplogroups and sub-haplogroups but also the definition of their distinguishing mutations (**Figure 1**). These include some diagnostic markers that are located in COI and ND5 partial sequences whose variation has been extensively assessed by published studies and can be retrieved from GenBank (**Table 2**).

**FIGURE 3:**
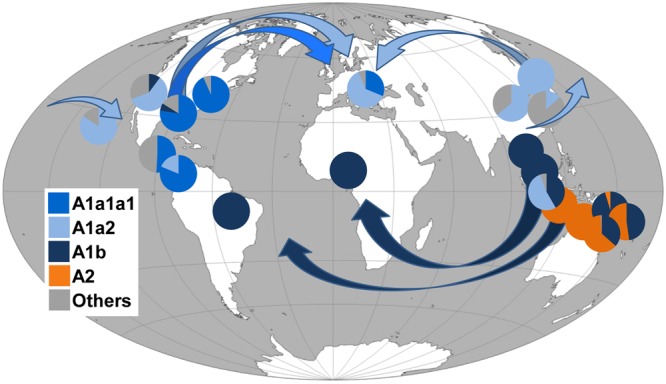
**Frequency (%) of *A. albopictus* mtDNA haplogroups in worldwide populations and possible diffusion routes.** Arrows indicate possible diffusion routes from the native home-range (South-East Asia) and subsequent dispersals. Populations and corresponding frequency values are listed in **Table 2**. Note that the Chinese laboratory strain was not included. The original world map is from the website (http://www.freeworldmaps.net).

By surveying these sequences for the presence or absence of these mutations, we were able to determine the most likely haplogroup affiliation for most of the 1170 tiger mosquito mtDNAs from populations worldwide. **Table 2** reports the haplogroup frequencies for A1a1a1, A1a2, A1b, A2, and A3 obtained from this survey, as well as a category termed “others” that includes mtDNAs that we could not classify and might encompass haplogroups not represented in our phylogeny. **Figure 3** provides an overview of the worldwide spatial distribution of these haplogroups.

## Discussion

### Geographical Distribution of *A. albopictus* mtDNA Haplogroups

Phylogenetic analyses revealed that our *A. albopictus* mitogenomes cluster into the three main haplogroups A1, A2, and A3. Intriguingly, the population screening of the COI mutations (1503, 1578C, 1676C, 1704, 1964) found in mitogenome #27 from Taiwan, the only one belonging to haplogroup A3 in our phylogeny, did not reveal any match in the 1170 tiger mosquito mtDNAs from worldwide populations, not even in the population sample (*N* = 30) from Taiwan (**Table 2**). This suggests that *A. albopictus* mosquitoes with A3 mtDNAs were probably not involved in the recent worldwide spread of the species, raising the possibility that this haplogroup might be rare and/or with a restricted geographical distribution.

In contrast, the survey for COI mutations characterizing the Philippine mitogenomes #21-25 allowed the identification of many other mtDNAs belonging to haplogroup A2 (**Table 2**), but all in Insular Southeast Asia, suggesting that this haplogroup might be typical and possibly limited to the Philippines, Indonesia, Papua New Guinea, and Northern Australia (**Table 2**). Therefore, haplogroup A2 appears to have played a role in the spread of *A. albopictus* from South-East Asia (**Figure 3**) restricted to the context of Oceania. Indeed *A. albopictus* is thought to have spread from Indonesia by human-mediated transportation (Beebe et al., 2013). The low frequency of A2 observed on the Papua New Guinean mainland further supports this scenario, whereas the presence of both A1b and A2 mtDNAs along the North Australian border (**Table 2**) suggests multiple arrivals from distinct geographical sources (Beebe et al., 2013).

**Figure 3** shows that, in contrast to the situation described above, members of the other three Asian haplogroups A1a1, A1a2, and A1b are detected in many adventive populations worldwide. This finding identifies these as the Asian mtDNA lineages mainly associated with the recent global spread.

As for haplogroup A1a1, seen in the Japanese mitogenome #26, it is widely distributed in Italy (**Figure 1**) and shows high frequencies in Central America and Eastern USA. Haplogroup A1a2 is present with frequencies higher than 50% in Japan, Southern China, Singapore, Hawaii, California and Italy, whereas A1b is fixed or almost fixed in Thailand, Malaysia, the Papua New Guinea mainland, Cameroon, and Brazil, but present at much lower frequencies also in California and Texas (**Table 2**). Even though these geographical distributions are based on the limited population sampling reported in **Table 2**, some preliminary conclusions can be drawn.

It appears that the ancestral homeland of haplogroup A1a2 might have been a temperate area, possibly Japan or Northern Asia, rather than the tropical range, in agreement with early allozyme studies (Kambhampati et al., 1991). In contrast haplogroup A1b appears to mainly characterize the tropical belt (**Figure 3**). This may imply that genetic and physiological traits make populations with A1b most suited to the colonization of tropical areas (Birungi and Munstermann, 2002; Kamgang et al., 2011). In our phylogeny the Brazilian A1b mitogenome (#9) harbors a mutational motif that includes the transition at np 7210 in the ND5 gene, also found in all Brazilian samples retrieved from the literature and already identified as a marker for Brazilian *A. albopictus* (Birungi and Munstermann, 2002). The same transition was found in two ND5 haplotypes retrieved from the literature, one from Phuntsholing in Southern Bhutan (JQ436953) and one from Chiang Mai in Thailand (JQ436956; Porretta et al., 2012), suggesting a probable route of invasion from Indochina (Goubert et al., 2016). Instead the absence of this transition in the samples from Cameroon (**Table 2**) suggests that A1b mtDNAs arrived in Cameroon from a different tropical source. Interestingly, the absence of photoperiodic diapause in Brazilian mosquitoes supports their origin in tropical Asia, while the diapause in US populations is in agreement with the scenario of an ancestry in the Asian temperate regions (Mousson et al., 2005; Urbanski et al., 2010).

As for haplogroup A1a1, its distribution could not be fully assessed because of the lack of informative marker mutations. However, the absence of the COI transitions at nps 1536 and 2165 distinguishes the members of its main sub-branch, A1a1a1 (**Figure 1**), from all other mitogenomes in the phylogeny. The survey of these transitions in published data sets revealed that haplogroup A1a1a1 is the most common in Costa Rica, Panama, Texas, and New Jersey (**Figure 3**) and widespread in Italy (**Table 2**). This distribution suggests that A1a1a1 and/or its derivatives A1a1a1a and A1a1a1a1 arose recently in an adventive non-Asian population, probably from an ancestral Japanese source, and reached a high frequency because of genetic drift or founder events. From the earliest adventive non-Asian population(s) they then further spread to other distant regions. Such a possibility is in agreement with some previous observations, in particular with the suggestion that Italian tiger mosquitoes, whose first presence was documented in Northern Italy in 1990 (Sabatini et al., 1990), have a dual origin: a possibly direct Northern American source and a probably indirect (through Albania and Greece) Eastern Asian source, with the former related to the international trade of used tires from the eastern coast of the US (Dalla Pozza et al., 1994; Dritsou et al., 2015).

An origin of haplogroup A1a1a1 and/or its derived sub-branches A1a1a1a and A1a1a1a1 in North America and a subsequent arrival to Italy from the US of multiple haplotypes is supported by (i) the detection of four partial sequences from Texas with the transition at np 1823 (Zhong et al., 2013), which defines sub-branch A1a1a1a1, and (ii) our findings that mitogenomes #4 and #5, the first from Virginia and the second from Italy, are identical, and that mitogenomes #2 and #3, again one from Virginia and one from Italy, are closely related (**Figure 1**). Finally, the scenario that the ancestral source of A1a1a1 might be a northern temperate area such as Japan is further supported by the presence of the COI transitions 1536 and 2435, which characterize mitogenome #26, in five published mtDNAs from Kyoto (JQ004524; Xu and Fonseca, 2011), and is in agreement with allozyme data that have highlighted genetic links between North American, Italian and Japanese populations (Urbanelli et al., 2000).

## Conclusion

Through our analyses, based on complete coding regions, the phylogeny of the *A. albopictus* mitogenomes was charted, and the most likely Asian sources of some adventive populations were identified. The worldwide spread of *A. albopictus* appears to be associated with three mtDNA haplogroups (A1a1, A1a2, and A1b), differently distributed in Asian populations living in temperate and tropical regions, whereas a fourth Asian haplogroup (A2) appears to be restricted to Insular South-East Asia. These ancestral genetic sources now coexist (and interbreed) in many of the recently colonized areas. This occurs not only in the field but also in the laboratory as attested by our detection of both A1a1a1 and A1a2a1 mitogenomes in the Rimini maintained strain, thus creating novel genomic combinations that might be one of the causes of the continuous and apparently growing capability of *A. albopictus* to expand its geographical range.

Note that fine scale mitogenome surveys, encompassing multiple specimens from a wide range of East Asian populations might prove to be an essential pre-requisite to controlling the spread of this mosquito and limiting its social, medical, and economic implications. With a precise identification of the source populations in Asia it will become possible to evaluate the extent and nature of their nuclear genome diversity and the possible selective advantages (e.g., production of cold or desiccation -resistant eggs, zoophilic versus anthropophilic changes in feeding behavior) relative to other Asian *A. albopictus* populations that, by contrast, have not spread.

## Author Contributions

VB, PG, AT, and AO conceived and designed the experiments. VB, PG, and SB performed the experiments. VB, PG, MRC, AA, LMG, AT, and AO analyzed the data. PG, PAJ, X-GC, AA, OS, LMG, ARM, GG, AT, and AO contributed reagents/materials/analysis tools. VB, PG, GG, AT, and AO wrote the manuscript. All authors contributed to interpretation of data, reviewed and approved the manuscript.

## Conflict of Interest Statement

The authors declare that the research was conducted in the absence of any commercial or financial relationships that could be construed as a potential conflict of interest.
