# Supplementary material for: The Worldwide Spread of the Tiger Mosquito as Revealed by Mitogenome Haplogroup Diversity
Source: Front Genet. 2016 Nov 23;7:208. doi: 10.3389/fgene.2016.00208 (PMC5120106; doi:10.3389/fgene.2016.00208)
Supplement: Supplementary file 1 [file Data_Sheet_1.DOCX]

Supplementary Material

The worldwide spread of the tiger mosquito as revealed by mitogenome haplogroup diversity

Vincenza Battaglia^1^, Paolo Gabrieli^1^, Stefania Brandini^1^, Marco R. Capodiferro^1^, Pio A. Javier^2^, Xiao-Guang Chen^3^, Alessandro Achilli^1^, Ornella Semino^1^, Ludvik M. Gomulski^1^, Anna R. Malacrida^1^, Giuliano Gasperi^1*^, Antonio Torroni^1*^, and Anna Olivieri^1*^

*** Correspondence:** Anna Olivieri: anna.olivieri@unipv.it, Antonio Torroni: antonio.torroni@unipv.it, Giuliano Gasperi: [giuliano.gasperi@unipv.it](mailto:giuliano.gasperi@unipv.it)

**Supplementary information for Figure 1.** There are 347 coding-region mutations (nps 1-14896) as well as 16 control-region mutations (14897-17150) that separate mitogenome #27 from the A1'2 node in Figure 1. The location of these mutations is indicated by the asterisk. These control-region mutations were included because they were assessed in all mitogenomes (except #9 and #13). The 363 mutations are listed below and are numbered relative to mitogenome #1. They are transitions unless a base is explicitly indicated for transversions (to A, G, C, or T) or a suffix for indels (.1, d). The suffix @ marks back mutations and the suffix "inv" indicates the inversion of the segment comprised between the specified positions.

Coding-region mutations: 143.1A 496 1423.1C 1503 1578C 1676C 1704 1964 2939 2982 2983 2984 3038.1TTAA 3196 3751A 3759T 3774-81inv 3992G 4085 4443 4470 5167C 5168T 5179 5187T 5197 5202 5204A 5216A 5222A 5244 5246A 5264 5289T 5290C 5294T 5303A 5311A 5313 5355A 5378A 5396 5404 5408T 5458A 5481 5483A 5514d 5593C 6191C 6511A 6512A 6514 6518T 6520A 6528G 6535C 6536C 6547A 6548 6550T 6551 6552 6556 6559C 6565T 6573T 6583G 6584 6586A 6589A 6591A 6596 6602A 6610A 6619A 6620A 6621 6624A 6625A 6636T 6643A 6650C 6651 6654G 6664 6667 6670A 6671A 6673A 6676G 6684A 6685T 6687T 6691A 6700 6708A 6712 6714A 6717 6719T 6736T 6737G 6738 6739 6757 6761 6772G 6775T 6784 6792C 6794 6803 6820A 6821 6822 6823 6826C 6830G 6831 6841A 6843A 6846G 6852T 6853 6854C 6855G 6857C 6868A 6892G 6898T 6910A 6919T 6951C 6957 6964A 6974 6994C 7003 7006 7020 7021C 7039A 7044 7054A 7060T 7078A 7079 7085 7086 7087 7093T 7096T 7118 7123 7132A 7133 7134A 7138T 7156G 7158A 7168T 7180A 7198 7213A 7222A 7234C 7249A 7267 7294 7303A 7315T 7320A 7324 7327A 7329 7345T 7349T 7350T 7351 7352C 7353A 7360A 7362 7366A 7369 7371 7378 7391A 7392T 7665A 8032T 8055G 8056T 8057A 8077C 8078A 8079T 8089 8099d 8101T 8102 8103C 8137.1A 8178 8348 8469 8934 8952T 8958 8959C 8965A 8970T 8971A 8973A 8974A 8975A 8976T 8978T 8979T 8980A 8981A 8985 8986C 8989T 8990A 8992T 8994T 8995A 8996A 8999A 9000A 9001 9006T 9007T 9008 9009T 9010A 9011 9013A 9014G 9016A 9018C 9030 9042 9048G 9060G 9081C 9100 9102G 9104T 9267 9274A 9324 9704 9808d 9809C 9817 9831 9832 9926.1T 9950 9951 9952T 9991 9992G 10031T 10034 10036G 10092T 10099A 10100T 10101 10121 10133T 10136 10152T 10155 10158A 10160A 10167C 10173A 10175T 10177G 10178C 10179A 10182T 10183A 10185T 10186 10187 10188A 10189 10193 10194C 10195A 10196T 10198A 10200T 10203A 10213A 10808 10858A 10867A 10870 10876G 10917A 10918T 10922C 10982A 10988T 10989A 10990 11635d 11829 12158A 12266@ 12299C 12407 12440A 12461 12532 12616T 12617-22d 12668G 12672T 12693d 13430d 13463d 13470.1T 13655G 13672 13880G 14027d 14114.1C 14121.1G 14131.1T 14143.1T 14164C 14179G 14180.1T 14182.1A 14183G 14548d 14769.1TA 14857.

Control-region mutations: 14953 14959G 14962d 14969 15106A 15142d 15159 15199d 15349 15476 (not assessed in samples #8, #12, #14 and #19) 15482d (not assessed in samples #8, #12, #14 and #19) 16759G 16801 16856.2TT 17119G 17123. Of these, three (14969, 15159, 15349) are recurrent mutations, ten (15106A, 15142d, 15199d, 15476 15482d 16759G 16801 16856.2TT 17119G 17123) are shared by mitogenomes #17 and #27 and three (14953, 14959G, 14962d) are private mutations of mitogenome #27.

Note that for mitogenomes #9 and #13 (in squared boxes), the sequence variation was assessed only partially for the coding region and not at all for the control region. As for the coding region, mitogenome #9 lacks sequence data for nucleotide positions (nps) 1-318, 2863-2916, 4665-5731, 6617-7033, 8825-13183 and 14115-14896, whereas mitogenome #13 lacks information encompassing nps 3520-10533 and 11753-14597.

**Supplementary Table 1.** Amplicons and oligonucleotide pairs used to amplify the *A. albopictus* mitogenome in four overlapping PCR fragments (protocol I).

|  | **PCR ID Number** | **Fragment Length (bp)** | **Oligonucleotides** | | | | | | |
| --- | --- | --- | --- | --- | --- | --- | --- | --- | --- |
|  |  |  | **Name^a^** | **5' np^b^** | **3' np^b^** | **Length (nt)** | | **Sequence (5’→3’)** | **Melting Temperature (°C)^d^** |
| **Coding region** | 1 | 8,602 | 274F | 274 | 295 | 22 | AGCTAACTCTTGATTAGGGGCA | | 58.88 |
|  |  |  | 8875R | 8875 | 8856 | 20 | TGTTGAGGCACCTGTTTCAG | | 58.32 |
|  | 2 | 6,303 | 8415F | 8415 | 8434 | 20 | TTAAAGTCGGAGGAGCAGCT | | 58.73 |
|  |  |  | 14717R | 14717 | 14698 | 20 | AAATTTGTGCCAGCTACCGC | | 59.76 |
| **Control region** | 3 | ~1,700-2,500 | 14534F | 14534 | 14552 | 20 | AGGGTATCTAATCCTAGTTT^c^ | | 49.46 |
|  |  |  | 16267R | 16267 | 16249 | 19 | CTATGGGTCCTAAATGAAG | | 49.44 |
|  | 4 | ~819-903 | 16261F | 16261 | 16285 | 25 | CCCATAGGCTTATAAATACTTCACT | | 56.22 |
|  |  |  | 414R | 414 | 394 | 21 | GAAGAAGCAAAGGCTTGAACT | | 57.00 |

^a^ Oligonucleotide codes refer to the nucleotide position of the initial (5’) base: F, forward; R, reverse.

^b^ Nucleotide positions correspond to the *A. albopictus* Reference Sequence [NC006817].

^c^ Primer from Xu and Fonseca, 2011; the *A. albopictus* Reference Sequence [NC006817] harbors an A-deletion at the underlined nucleotide position.

^d^ The annealing temperature was 59°C for PCRs #1-2 (long PCR fragments), 54°C for PCR #3 and 55°C for PCR #4.

**Supplementary Table 2.** Amplicons and oligonucleotide pairs used to amplify the *A. albopictus* mtDNA coding region in nine overlapping PCR fragments (protocol II).

| **PCR ID Number** | **Fragment Length (bp)** | **Oligonucleotides** | | | | | |
| --- | --- | --- | --- | --- | --- | --- | --- |
|  |  | **Name^a^** | **5'np^b^** | **3'np^b^** | **Length (nt)** | **Sequence (5’→3’)** | **Melting Temperature (°C)**^f^ |
| 1 | 1,493 | 274F | 274 | 295 | 22 | AGCTAACTCTTGATTAGGGGCA | 58.88 |
|  |  | 1766R | 1766 | 1747 | 20 | GCAGCAGTGTTAAAGAGGGG | 58.83 |
| 2 | 2,159 | 1423F | 1423 | 1443 | 20 | TCAGCCATTTAATCGCGACA^c^ | 57.98 |
|  |  | 3582R | 3582 | 3563 | 20 | TTAAACGTCCGGGAGTAGCA | 58.74 |
| 3 | 2,167 | 3272F | 3272 | 3294 | 23 | TGCCTTTCCTTCTTTACGACTTT | 58.54 |
|  |  | 5438R | 5438 | 5416 | 23 | CTGCTGCTTCAAAACCAAAATGA | 58.88 |
| 4 | 2,181 | 5269F | 5269 | 5298 | 22 | CGAAGCACCTTTTACAATTGCA^d^ | 58.36 |
|  |  | 7448R | 7448 | 7424 | 25 | TACTCCTGTTTCTGCTTTAGTTCAT | 58.23 |
| 5 | 1,754 | 7125F | 7125 | 7147 | 23 | TTGTGAATAATTACCCCAGCACA^e^ | 58.33 |
|  |  | 8875R | 8875 | 8856 | 20 | TGTTGAGGCACCTGTTTCAG | 58.32 |
| 6 | 1,647 | 8799F | 8799 | 8821 | 23 | CCCGTAATAAACCATATCCTCCC | 58.16 |
|  |  | 10445R | 10445 | 10425 | 21 | TTTATTGGTCGTAATGGGCCA | 57.63 |
| 7 | 1,816 | 9985F | 9985 | 10007 | 23 | AAACATCTGCTTGCTATAGGTCT | 57.63 |
|  |  | 11800R | 11800 | 11783 | 18 | GGGACTTTGCCTCGGTTT | 57.51 |
| 8 | 1,819 | 11460F | 11460 | 11479 | 20 | TGAATTGGAGCTCGACCTGT | 59.02 |
|  |  | 13278R | 13259 | 13288 | 20 | AAGGGCCGCAGTATTTTGAC | 58.83 |
| 9 | 1,925 | 12793F | 12793 | 12812 | 20 | CGCCGGTTTGAACTCAGATC | 59.28 |
|  |  | 14717R | 14717 | 14698 | 20 | AAATTTGTGCCAGCTACCGC | 59.76 |

^a^ Oligonucleotide codes refer to the nucleotide position of the initial (5’) base: F, forward; R, reverse.

^b^ Nucleotide positions correspond to the *A. albopictus* Reference Sequence [NC006817].

^c^ The *A. albopictus* Reference Sequence [NC006817] harbors a C-insertion between the underlined nucleotide positions.

^d^ The *A. albopictus* reference sequence [NC006817] harbors C>T at the underlined nucleotide position.

^e^ The *A. albopictus* reference sequence [NC006817] harbors G>A, T>A, A>G, C>A and A>T at the underlined nucleotides, respectively.

^f^ The annealing temperature for all PCR reactions was 55°C.

**Supplementary Table 3.** Oligonucleotides used for sequencing the *A. albopictus* mtDNA coding region (protocol I).

| **Template PCR ID Number^a^** | **Sequencing Oligonucleotides** | | | | | | |  |
| --- | --- | --- | --- | --- | --- | --- | --- | --- |
|  | | **Name^b^** | **5'np^c^** | **3'np^c^** | **Length (nt)** | **Sequence (5’→3’)** | **Melting Temperature (°C)** | |
| 1 | | 274F^d^ | 274 | 295 | 22 | AGCTAACTCTTGATTAGGGGCA | 58.88 | |
| 1 | | 1452F | 1452 | 1476 | 25 | GGTCAACAAATCATAAAGATATTGG^e^ | 54.80 | |
| 1 | | 1766R | 1766 | 1747 | 20 | GCAGCAGTGTTAAAGAGGGG | 58.83 | |
| 1 | | 2027F | 2027 | 2046 | 20 | CCCGTATTAGCCGGAGCTAT^e^ | 58.82 | |
| 1 | | 2867F | 2867 | 2887 | 21 | CGAACTCCTTCTTTCCCCATA | 57.09 | |
| 1 | | 4063F | 4063 | 4085 | 23 | TGACCCTTCAACTACTATTTTCA | 55.26 | |
| 1 | | 4233R | 4233 | 4214 | 20 | CCATTTGGCCCTAAAAGAGT | 55.59 | |
| 1 | | 4728F | 4728 | 4747 | 20 | CACATGCAAATCACCCTTTT | 55.02 | |
| 1 | | 5685F | 5685 | 5704 | 20 | CCCCTTTGAATGTGGATTTG | 54.80 | |
| 1 | | 5910R | 5910 | 5890 | 21 | TGCTCCTTGATTTCATTCATG | 54.52 | |
| 1 | | 6287F | 6287 | 6309 | 23 | AATAATTTCCCCAACATCTTCAA | 54.46 | |
| 1 | | 6992F | 6992 | 7013 | 22 | ATCCTTAGAATAAAATCCCGCT^f^ | 55.00 | |
| 1 | | 7441R | 7441 | 7419 | 23 | GTTTCTGCTTTAGTTCATTCTTC | 54.27 | |
| 1 | | 7638F | 7638 | 7658 | 21 | CCAATTCGATTAGAAAGAGCA | 57.15 | |
| 1 | | 8415F^d^ | 8415 | 8434 | 20 | TTAAAGTCGGAGGAGCAGCT | 58.73 | |
| 2 | | 8658F | 8658 | 8677 | 20 | GAGCTACAGAAGAATAAGCA | 52.44 | |
| 2 | | 9085F | 9085 | 9104 | 19 | TGCTTGTAATCGTTCTGGT^g^ | 54.41 | |
| 2 | | 9986F | 9986 | 10007 | 22 | AACATCTGCTTGCTATAGGTCT | 56.96 | |
| 2 | | 10425F | 10425 | 10444 | 20 | TGGCCCATTACGACCAATAA | 56.90 | |
| 2 | | 11799R | 11799 | 11782 | 18 | GGACTTTGCCTCGGTTTC | 56.01 | |
| 2 | | 11460F | 11460 | 11479 | 20 | TGAATTGGAGCTCGACCTGT | 59.02 | |
| 2 | | 12193F | 12193 | 12212 | 20 | CTACTGCTCGTAAACCCCCT | 58.81 | |
| 2 | | 12913F | 12913 | 12937 | 25 | GAACTCTAAAAAAAAATTACGCTGT | 55.38 | |
| 2 | | 13288F | 13288 | 13307 | 20 | TCAGTGGGCAGGTTAGACTT | 58.27 | |
| 2 | | 13820F | 13820 | 13839 | 20 | ACCCTGATACACAAGGTACA | 55.46 | |
| 3 | | 14534F^d^ | 14534 | 14552 | 20 | AGGGTATCTAATCCTAGTTT^h^ | 49.46 | |
| 4 | | 16261F^d^ | 16261 | 16285 | 25 | CCCATAGGCTTATAAATACTTCACT | 56.22 | |
| 4 | | 414R^d^ | 414 | 394 | 21 | GAAGAAGCAAAGGCTTGAACT | 57.00 | |

^a^ Template PCR ID Numbers refer to those in Supplementary Table S1.

^b^ Oligonucleotide codes refer to the nucleotide position of the initial (5’) base: F, forward; R, reverse.

^c^ Nucleotide positions correspond to the *A. albopictus* Reference Sequence [NC006817].

^d^ Primers used for PCR reactions (Table S1).

^e^ Primer from Zhong et al., 2013; the *A. albopictus* Reference Sequence [NC006817] harbors two G>T at the underlined nucleotides.

^f^ The *A. albopictus* reference sequence [NC006817] harbors A>C, T>C and C>T at the underlined nucleotides, respectively.

^g^ The *A. albopictus* Reference Sequence [NC006817] harbors an A-insertion between the underlined nucleotides.

^h^ Primer from Xu and Fonseca, 2011; the *A. albopictus* Reference Sequence [NC006817] harbors an A-deletion at the underlined nucleotide position.

**Supplementary Table 4.** Oligonucleotides used for sequencing the *A. albopictus* mtDNA coding region (protocol II).

| **Template PCR ID Number^a^** | **Sequencing Oligonucleotides** | | | | | |
| --- | --- | --- | --- | --- | --- | --- |
|  | **Name^a^** | **5'np^b^** | **3'np^b^** | **Length (nt)** | **Sequence (5’→3’)** | **Melting Temperature (°C)** |
| 1 | 274F^c^ | 274 | 295 | 22 | AGCTAACTCTTGATTAGGGGCA | 58.88 |
| 4^d^ | 414R^c^ | 414 | 394 | 21 | GAAGAAGCAAAGGCTTGAACT | 57.00 |
| 1 | 1766R^c^ | 1766 | 1747 | 20 | GCAGCAGTGTTAAAGAGGGG | 58.83 |
| 2 | 1452F | 1452 | 1476 | 25 | GGTCAACAAATCATAAAGATATTGG^e^ | 54.80 |
| 2 | 2027F | 2027 | 2046 | 20 | CCCGTATTAGCCGGAGCTAT^e^ | 58.82 |
| 2 | 2867F | 2867 | 2887 | 21 | CGAACTCCTTCTTTCCCCATA | 57.09 |
| 3 | 4233R | 4233 | 4214 | 20 | CCATTTGGCCCTAAAAGAGT | 55.59 |
| 3 | 4063F^c^ | 4063 | 4085 | 23 | TGACCCTTCAACTACTATTTTCA | 55.26 |
| 3 | 4728F | 4728 | 4747 | 20 | CACATGCAAATCACCCTTTT | 55.02 |
| 3 | 5143F | 5143 | 5164 | 22 | AGGAGTTACTGTTACATGGGCT | 58.82 |
| 4 | 5685F | 5685 | 5704 | 20 | CCCCTTTGAATGTGGATTTG | 54.80 |
| 4 | 5910R | 5910 | 5890 | 21 | TGCTCCTTGATTTCATTCATG | 54.52 |
| 4 | 6287F | 6287 | 6309 | 23 | AATAATTTCCCCAACATCTTCAA | 54.46 |
| 4 | 6992F | 6992 | 7013 | 22 | ATCCTTAGAATAAAATCCCGCT^f^ | 55.00 |
| 5 | 7125F^c^ | 7125 | 7147 | 23 | TTGTGAATAATTACCCCAGCACA^g^ | 58.33 |
| 5 | 7638F | 7638 | 7658 | 21 | CCAATTCGATTAGAAAGAGCA | 57.15 |
| 5 | 8415F^c^ | 8415 | 8434 | 20 | TTAAAGTCGGAGGAGCAGCT | 58.73 |
| 6 | 8798F^c^ | 8798 | 8819 | 22 | ACCCGTAATAAACCATATCCTC | 55.12 |
| 6 | 9085F | 9085 | 9104 | 19 | TGCTTGTAATCGTTCTGGT^h^ | 54.41 |
| 6 | 9564F | 9564 | 9583 | 20 | AGACAACCCTAATACCCCTT | 55.08 |
| 6 | 9986F | 9986 | 10007 | 22 | AACATCTGCTTGCTATAGGTCT | 56.96 |
| 7 | 10425F | 10425 | 10444 | 20 | TGGCCCATTACGACCAATAA | 56.90 |
| 7 | 11799R | 11799 | 11782 | 18 | GGACTTTGCCTCGGTTTC | 56.01 |
| 8 | 11460F^c^ | 11460 | 11479 | 20 | TGAATTGGAGCTCGACCTGT | 59.02 |
| 8 | 12193F | 12193 | 12212 | 20 | CTACTGCTCGTAAACCCCCT | 58.81 |
| 8 | 12913F | 12913 | 12937 | 25 | GAACTCTAAAAAAAAATTACGCTGT | 55.38 |
| 9 | 13288F | 13288 | 13307 | 20 | TCAGTGGGCAGGTTAGACTT | 58.27 |
| 9 | 13820F | 13820 | 13839 | 20 | ACCCTGATACACAAGGTACA | 55.46 |
| 9 | 14534F^c^ | 14534 | 14552 | 20 | AGGGTATCTAATCCTAGTTT^i^ | 49.46 |

^a^ Template PCR ID Numbers refer to those in Supplementary Table S2.

^b^ Oligonucleotide codes refer to the nucleotide position of the initial (5’) base: F, forward; R, reverse.

^c^ Primers used also for PCR reactions (Table S2).

^d^ Template PCR ID Number refer to those in Supplementary Table S1.

^e^ Primer from Zhong et al., 2013; the *A. albopictus* Reference Sequence [NC006817] harbors two G>T at the underlined nucleotides.

^f^ The *A. albopictus* reference sequence [NC006817] harbors A>C, T>C and C>T at the underlined nucleotides, respectively.

^g^ The *A. albopictus* reference sequence [NC006817] harbors G>A, T>A, A>G, C>A and A>T at the underlined nucleotides, respectively.

^h^ The *A. albopictus* Reference Sequence [NC006817] harbors an A-insertion between the underlined nucleotide positions.

^i^ Primer from Xu and Fonseca, 2011; the *A. albopictus* Reference Sequence [NC006817] harbors an A-deletion at the underlined nucleotide.

**Supplementary Table 5.** Oligonucleotide pairs used to amplify the segment of the *A. albopictus* control region containing type I tandem repeats in two overlapping PCR fragments.

| **PCR ID Number** | **Oligonucleotides** | | | | | |
| --- | --- | --- | --- | --- | --- | --- |
|  | **Name^a^** | **5' np^b^** | **3' np^b^** | **Length (nt)** | **Sequence (5’→3’)** | **Melting Temperature (°C)** |
| I | 14534F^c^ | 14534 | 14552 | 20 | AGGGTATCTAATCCTAGTTT^d^ | 49.46 |
|  | 15807R^e,f^ | 15788 | 15807 | 20 | AGGGGTTAATTTAATAAGTT | 46.84 |
| II | 15016F^f^ | 15016 | 15035 | 20 | TTCCTAGAAGCATCAAACTT | 52.07 |
|  | 16268R | 16246 | 16268 | 23 | CCTATGGGTCCTAAATGAAGAAA | 55.70 |

^a^ The oligonucleotide codes refer to the nucleotide position of the initial (5’) base: For, forward; Rev, reverse.

^b^ Nucleotide positions correspond to the *A. albopictus* Reference Sequence [NC006817].

^c^ Primer from Zhong et al., 2013.

^d^ The *A. albopictus* Reference Sequence [NC006817] harbors an A-deletion at the underlined nucleotide position.

^e^ Primer from Xu and Fonseca, 2015.

^f^ Primers priming in the tandemly repeated elements.

**Supplementary Figure 1.** *A. albopictus* phylogeny obtained by using Maximun Likelihood (ML). As described in Material and Methods, this tree was obtained by using PAMLX (Yang, 2007). This method does not calculate bootstrap values at tree nodes. However, to further assess the reliability of the A1 node that in the MP analysis showed a bootstrap value of 60% (Figure 1), we calculated the bootstrap proportions between our ML tree and an alternative one without the A1 node. The analysis was performed with PAMLX using the RELL method (Kishino and Hasegawa, 1989), as well as the method of Shimodaira and Hasegawa (1999). The obtained value was 0.979.

**Supplementary References**

Kishino, H., Hasegawa, M. (1989). Evaluation of the maximum likelihood estimate of the evolutionary tree topologies from DNA sequence data, and the branching order in hominoidea. *J. Mol. Evol.* 29, 170-179.

Shimodaira, H. and Hasegawa, M. (1999). Multiple comparisons of log-likelihoods with applications to phylogenetic inference. *Mol. Biol. Evol.* 16, 1114-1116.

Xu, J. and Fonseca, D.M. (2011) One-way sequencing of multiple amplicons from tandem repetitive mitochondrial DNA control region. *Mitochondrial DNA* 22, 155-158. doi: 10.3109/19401736.2011.636434

Yang, Z. (2007). PAML 4: Phylogenetic analysis by Maximum Likelihood. *Mol. Biol. Evol.* 24, 1586-1591. 10.1093/molbev/msm088

Zhang, H., Xing, D., Wang, G., Li, C. and Zhao, T. (2015) Sequencing and analysis of the complete mitochondrial genome of *Aedes albopictus* (Diptera: Culicidae) in China. *Mitochondrial DNA A DNA MappSeq. Anal.* 27, 2787-2788. doi: 10.3109/19401736.2015.1053067

Zhong, D., Lo E., Hu, R., Metzger, M.E., Cummings, R., Bonizzoni, M., et al. (2013) Genetic analysis of invasive *Aedes albopictus* populations in Los Angeles County, California and its potential public health impact. *PLoS One* 8:e68586. doi: 10.1371/journal.pone.0068586
